# Cannabis Glandular Trichomes: A Cellular Metabolite Factory

**DOI:** 10.3389/fpls.2021.721986

**Published:** 2021-09-20

**Authors:** Cailun A. S. Tanney, Rachel Backer, Anja Geitmann, Donald L. Smith

**Affiliations:** Department of Plant Science, Macdonald Campus, McGill University, Montreal, QC, Canada

**Keywords:** *Cannabis*, trichome, flower, metabolite, cannabinoid, terpene, inflorescence

## Abstract

*Cannabis* has been legalized for recreational use in several countries and medical use is authorized in an expanding list of countries; markets are growing internationally, causing an increase in demand for high quality products with well-defined properties. The key compounds of *Cannabis* plants are cannabinoids, which are produced by stalked glandular trichomes located on female flowers. These trichomes produce resin that contains cannabinoids, such as tetrahydrocannabinolic acid and cannabidiolic acid, and an array of other secondary metabolites of varying degrees of commercial interest. While growers tend to focus on improving whole flower yields, our understanding of the “goldmines” of the plant – the trichomes – is limited despite their being the true source of revenue for a multi-billion-dollar industry. This review aims to provide an overview of our current understanding of cannabis glandular trichomes and their metabolite products in order to identify current gaps in knowledge and to outline future research directions.

## Introduction

Trichomes are formed on the plant surface across a range of taxonomically disparate species, providing a variety of functions and benefits to the plant. These can include simple tasks, such as affecting leaf temperature and photosynthesis, or more complicated functions, such as pest-deterrence *via* their physical structures or production of compounds (Wagner, 1991; Hare et al., 2003). Glandular trichomes are of particular commercial interest as they are one of the key plant structures that produce essential oils – an industry valued at 18.62 billion USD in 2020 (Grand View Research, 2020). Other oil-producing plant structures are internal glands and other trichome types, some of which are capable of producing resinous secretions. Trichome morphology is highly variable both among plant species and within the plant itself (Sangwan et al., 2001). In *Cannabis sativa* L. (hereafter, cannabis), stalked glandular trichomes are the trichome morph that produces substances of economic value (Fairbairn, 1972; Sirikantaramas et al., 2005). These trichomes develop a secretory cavity between secretory disk cells and the cuticle where secondary metabolites, including cannabinoids and terpenes, are deposited and stored (Kim and Mahlberg, 1991, 1997; Sirikantaramas et al., 2005; Marks et al., 2009). Though there are a variety of other trichome morphs found across the cannabis plant, they are beyond the scope of this review.

**Figure 1:**
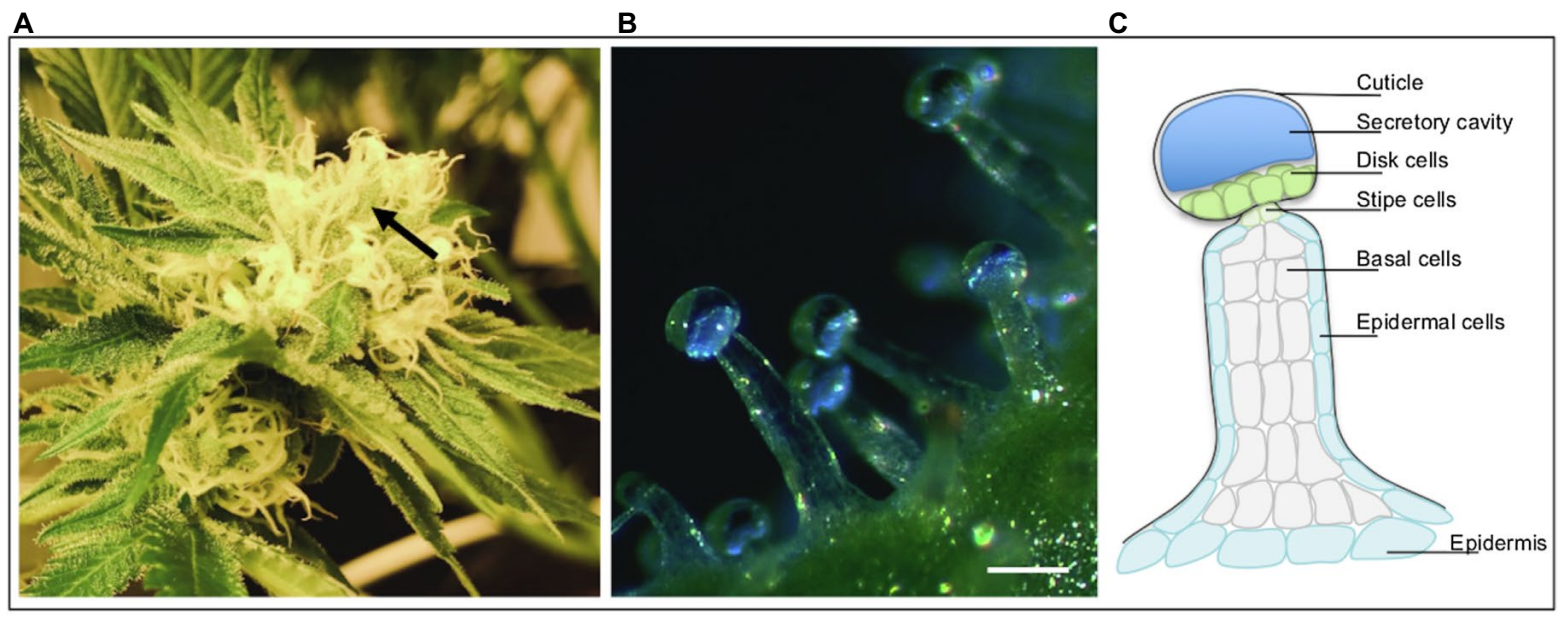
Cannabis (*Cannabis sativa* L.) inflorescence and trichomes. **(A)** An individual inflorescence, with majority of the organs covered in stalked glandular trichomes. Arrow indicates cluster of calyces and bracts covered with trichomes. **(B)** Dark field micrograph of stalked glandular trichomes protruding from calyx epidermis. Biosynthesis of secondary metabolites occurs in the secretory disk cells lining the base of the globular trichome head. The metabolites are stored in the clear subcuticular cavity above the secretory disk cells; this cavity will turn milky white to dark brown over the course of flower maturity. **(C)** Graphic illustration of stalked glandular trichome structure.

While male plants produce small amounts of cannabinoids, in cannabis cultivation, the primary products are the female flowers clustered in inflorescences (Ohlsson et al., 1971). Stalked glandular trichomes are primarily concentrated on the calyces and bracts (Figure 1A; Spitzer-Rimon et al., 2019; Leme et al., 2020) with populations extending to the inflorescence “sugar leaves”; these are the sites of accumulation for secreted metabolic products. These valuable secretions include tetrahydrocannabinolic acid (THCA), cannabidiolic acid (CBDA), terpenes, and flavonoids (ElSohly and Slade, 2005; Flores-Sanchez and Verpoorte, 2008). Cannabis plant morphology and cannabinoid profiles are influenced by genetics and the cultivation environment, highlighting the importance of controlled conditions for cannabis cultivation (Magagnini et al., 2018; Danziger and Bernstein, 2021a, b). With the gradual global increase in social and legal acceptance of cannabis, there has been considerable interest in producing consistent high-quality yields. In addition, as medicinal uses for cannabinoids are supported by peer-reviewed research and clinical trials, the global demand for medicinal cannabis products will continue to increase. This will create further pressure on growers to improve control over the concentration of specific cannabis metabolites and the associated cannabis genotypes. However, the genotypes and environmental conditions needed to obtain this level of precision remain poorly characterized. Ultimately, these elusive methods need to be centered around trichomes as the “factories” of the plant. Current efforts have focused on the effects of breeding and cultivar selection, industrial growing conditions, and fertilization methods on flower yield and cannabinoid profiles (Vanhove et al., 2011; Campiglia et al., 2017; Tang et al., 2017; Hawley et al., 2018; Janatová et al., 2018; Burgel et al., 2020; Saloner and Bernstein, 2021). However, as undefined cannabis plant material in pioneering research papers formed the backbone for future cannabis/cannabinoid research, comparing data with uniform standards is impossible. Thus, the need for systematically validating results of these papers and cannabis production “folklore” is paramount yet challenging due to the impact of genotype and growing environment. Regardless of these challenges, since trichomes are ultimately responsible for yield and quality control, it is necessary to advance our understanding of how they, specifically, are affected by these efforts, as well as to investigate new approaches to broaden the scope of possible cost-effective applications for improving yield.

## Trichome Profiles

### Trichomes Across the Plant Kingdom

Trichomes are found across the plant kingdom, displaying a stunning variety of shapes and properties. Glandular trichomes, which arise from the epidermis on vegetative and reproductive organs, can be generally divided into secretory and non-secretory types with the former being able to secrete substances (Tian et al., 2017). Both the morphologies and metabolic secretions of trichomes are consistent within a plant species, and some species have different trichome morphs on the same plant organ (Muravnik, 2020). Secreted compounds, including THCA (Sirikantaramas et al., 2005), can be toxic to plant cells; therefore, metabolite storage in the cavity of the glandular head affords protection to the plant (Sirikantaramas et al., 2008). While different glandular trichome morphs invoke different storage strategies, the architecture of the morph and cavity position in relation to the secretory cells determine secretion direction (Tissier et al., 2017). The molecular details surrounding the development of glandular trichomes and their secretions are beyond the scope of this article, and we refer to in-depth reviews by Muravnik (2020) and Tian et al. (2017).

Genomic studies are imperative to investigate the factors that influence trichome development in cannabis, both within and between cultivars. Trichome differentiation mechanisms have been investigated in *Arabidopsis thaliana*, with transcription factor (TFs) groups playing key roles in the transcriptional networks for trichome production and patterns (Tian et al., 2017). While genomic studies are available for other economic plants, including *Humulus lupulus* which belongs to the Cannabaceae family (Matoušek et al., 2016; Mishra et al., 2020), similar studies for cannabis are lacking despite their potentially important impacts for precise cannabis trichome control. Taking advantage of the genetic libraries available for related species with similar resin secretions will help guide these much-need studies (Braich et al., 2019; Zager et al., 2019; Liu et al., 2021).

### Key Cannabis Metabolites

The decades-long stigma surrounding cannabis has led to a variety of misconceptions surrounding the plant and its products regarding cannabinoid biosynthesis. While THCA and CBDA are the major cannabinoids produced by the plant, their degradation products, THC and CBD, are of great interest for their psychoactive and therapeutic effects. Additional cannabinoids are gradually gaining interest as their effects on the human body are beginning to be understood (ElSohly and Slade, 2005; Aizpurua-Olaizola et al., 2016; Andre et al., 2016). Fresh cannabis flower tissue contains relatively low levels of THC and CBD and higher levels of THCA and CBDA as the acid forms are converted to neutral forms *via* decarboxylation in post-harvest processing and storage; the rate of conversion is primarily dependent on temperature and light (Yamauchi et al., 1967). Metabolites, including cannabinoids, terpenes, and flavonoids, are formed within secretory disk cells that line the base of the glandular trichome head and stored in the subcuticular cavity (Figure 1B,C; Kim and Mahlberg, 1991, 1997). Within the cells, cannabinoid biosynthesis starts in the cytosol, moves to the plastid, and finishes with oxidocyclization in the apoplastic space; transport between these areas is not yet resolved (Gülck and Møller, 2020). A plethora of cannabinoids have been identified in recent years, bringing the total known number to just over 110, which can be divided into 11 subclasses (ElSohly and Gul, 2014; Andre et al., 2016; Hanuš et al., 2016; Berman et al., 2018). The biosynthesis pathways of the key cannabinoids, in particular THC and CBD, are described in detail in previous reviews (Gülck and Møller, 2020; Desaulniers Brousseau et al., 2021).

To date, over 120 terpenes have been identified in cannabis, which are broadly classified as monoterpenes and sesquiterpenes based on differences in their carbon skeletons (ElSohly and Slade, 2005; Degenhardt et al., 2009). Terpenes have a biosynthesis pathway similar to cannabinoids, and this process has been extensively reviewed (Booth et al., 2017; Desaulniers Brousseau et al., 2021). Terpenes impart floral aroma and flavor, making them important components for plant product applications, like essential oils, from many plant species. Terpene profiles vary among cannabis cultivars (Booth et al., 2017) and hemp oils containing more monoterpenes score better on olfactory evaluations than oils containing more sesquiterpenes while an oil containing a mix of both scored highest on scent tests (Mediavilla and Steinemann, 1997). Thus, the terpene composition of cannabis flowers at maturity can directly affect the olfactory quality of flower-based products and extracts, including essential oil-based goods.

Flavonoids are an additional major cannabis phytochemical group; however, this group of compounds has received less research focus compared to cannabinoids and terpenes. Similar to terpenes, flavonoids are found across a wide range of plant genera with a broad range of roles and benefits for the plant (Panche et al., 2016). There are over 20 identified flavonoids for cannabis, with three relatively unique compounds known as cannflavin A, B, and C (Bautista et al., 2021). The potential pharmaceutical uses of flavonoids, spanning from anti-inflammatories to anti-cancer therapies, are boosting interest in these compounds particularly as the entourage effects afforded by cannabis metabolite profiles become better understood (Tomko et al., 2020; Bautista et al., 2021). As flavonoids are produced primarily in cannabis leaves, not the inflorescences (Jin et al., 2020), the present article will focus on cannabinoids and terpenes.

## Cannabis Glandular Trichomes

Previously, three types of glandular trichomes on cannabis flowers were described – referred to as capitate-sessile, capitate-stalked, and bulbous – based on structural assessments by scanning electron microscopy (Hammond and Mahlberg, 1973). The trichomes were differentiated based on their morphology, where bulbous trichomes were small and low, sessile trichomes were comprised of a globular head on a very short stalk, and stalked trichomes had a larger globular head on a long stalk; of the three trichome types, stalked trichomes produce the greatest amount of cannabinoids (Hammond and Mahlberg, 1973; Mahlberg and Kim, 2004; Livingston et al., 2020). Unfortunately, this non-specific differentiation between trichome types led to misidentification of trichomes due to the similar appearance of sessile and stalked morphs (Dayanandan and Kaufman, 1976; Livingston et al., 2020). However, a recent study on trichome anatomy revealed that sessile trichomes on vegetative leaves consistently have exactly eight secretory disk cells while stalked glandular trichomes on mature flowers have 12–16; these numbers were consistent across hemp and drug-type varieties (Livingston et al., 2020). As sessile-presenting trichomes on immature cannabis flowers can contain more than eight disk cells and emit fluorescence at intermediate wavelengths, which true sessile trichomes cannot, sessile-presenting trichomes are now thought to be a precursor developmental stage of immature stalked trichomes (Livingston et al., 2020). These discoveries allow for improved accuracy of trichome classification during plant development, may provide more precise estimates of plant maturity and allow for identification of optimal points of metabolite production. This understanding further allows for greater accuracy when assessing the density of stalked glandular trichomes and the ability to predict mature flower trichome densities.

The causes of variable metabolite profiles found among varieties/genotypes and plant organs are genetic and environmental. For example, flowers sampled from the upper region of the plant produce significantly greater quantities of cannabinoids and terpenes than lower positions; light source and plant maturity are believed to be important factors influencing the concentration and/or amounts (Namdar et al., 2018; Eichhorn Bilodeau et al., 2019). Abiotic factors that influence cannabis growth are the same as those affecting other plant species, such as temperature, fertilization, photoperiod, and light intensity (Taschwer and Schmid, 2015; Conant et al., 2017; Pagnani et al., 2018; Bernstein et al., 2019; Eichhorn Bilodeau et al., 2019; Taghinasab and Jabaji, 2020). However, knowledge regarding how these factors influence growth and trichome formation is limited, with much work needed to produce scientific evidence to support links between metabolite production and environmental factors (Taghinasab and Jabaji, 2020). Research on cannabis is in the early stages, and future work is necessary to investigate signaling pathways that mediate the effect of external factors on metabolite production. Attention toward developing this area of cannabis research is increasing (Mudge et al., 2019; Aliferis and Bernard-Perron, 2020; Conneely et al., 2021).

### Potential Benefits of Cannabis Trichomes to the Plant

The exact benefit of cannabinoids and terpenes for the plant has yet to be discovered but several findings point to defense-related functions. This is consistent with a common role of trichomes in many plant species (Levin, 1973). Early studies have also hypothesized that THC protects against ultraviolet (UV) radiation, as cannabis plants produce significantly elevated levels of THC when exposed to higher levels of UVB radiation, possibly resulting in the development of geographical chemotypes (Pate, 1983). A recent study found that CBD could be a potential sunscreen additive as its application to human keratinocyte and melanocyte cells led to improved cell viability after exposure to UVB radiation, suggesting that cannabinoids protect cells against this type of potentially DNA-damaging radiation and supporting the geographical chemotype hypothesis (Gohad et al., 2020). These findings indicate that cannabinoids may be secreted and concentrated around flowers to protect the reproductive organs – and thereby the next generation – from the effects of sun damage; genotypes that originate from closer to the equator will produce higher levels of cannabinoids due to the higher incidence of UVB radiation in that region.

Terpenes may act as deterrents against herbivory, as the monoterpenes α-pinene and limonene repel insects are present in higher concentrations in flowers while sesquiterpenes, which are bitter to mammals, have greater concentrations in the lower leaves (Potter, 2009; Nerio et al., 2010; Russo, 2011). This apparent range of terpene profiles, dependent on organ and position, is in line with probable causes of damage, as insects would be more likely to damage the flowers and herbivorous mammals are likely to focus on the larger fan leaves. In addition, cannabinoids and terpenes can complement each other to provide plants with a complex defense mechanism against insects. The ratio of monoterpenes to sesquiterpenes determines cannabis resin viscosity while CBGA and THCA are toxic to insects. Altering the ratio of terpene types to increase viscosity can trap insects while CBGA and THCA induce apoptosis as shown on cultured insect cell lines, thus protecting the plant and critical tissues like flowers as they develop (Sirikantaramas et al., 2005; Russo, 2011). Terpenes and cannabinoids also interact after ingestion by animals as terpenes were shown to contribute to the affinity of THC to cannabinoid receptor 1 receptors in humans, among other effects (Russo and McPartland, 2001; Andre et al., 2016). The interactions between terpenes and cannabinoids are thus subject to ongoing investigations, not only to gain insight into the role of terpenes for plants, but also due to the potential therapeutic benefits which the medicinal cannabis sector could leverage.

The role of cannabinoids in biotic stress tolerance is consistent with their elevated concentration in flowers where trichome densities are highest. In addition to reducing the risk of pest-related damage, cannabinoids also have antimicrobial properties. Five key compounds [THC, CBD, cannabichromene (CBC), cannabigerol (CBG), and cannabinol (CBN)] and their acid precursor forms have significant antibacterial activity against several methicillin-resistant *Staphylococcus aureus* strains through bacterial membrane targeting (van Klingeren and ten Ham, 1976; Appendino et al., 2008; Farha et al., 2020). This suggests that cannabinoids, including those that are typically secreted in low concentrations, have a broad range of benefits, acting both within and outside the plant, particularly with regards to cannabinoid production in flowers when compared to the rest of the plant (Farha et al., 2020). However, while there is an increasing understanding of the defensive properties of the major metabolic products produced by cannabis, the lesser-known compounds must also be given attention. As there have been over 200 identified cannabinoid and terpene compounds combined, the costs for producing this vast number of secondary metabolites must be investigated to elucidate their individual benefits and roles in plant function. Transcriptomic studies into these lesser-known compounds and their expression in response to common stressors could provide an important start into answering these questions.

Overall, the range of potential benefits of these secondary metabolites strongly suggests that they play a key role in the general health and survival of cannabis plants and their progeny through a combination of factors. To corroborate this, genomics, transcriptomics, and metabolomics studies must be conducted to confirm hypothesized characteristics associated with various trichome morphs, their development patterns across different tissues, and their non-uniform metabolite secretions. Evidence is required to prove that these compounds are not simply by-products of other biological processes but truly have a primary role in defense mechanisms. To be meaningful, these studies should not only include cannabis cultivars that are the result of centuries of breeding, but also naturally occurring types that are not products of human selection activity, though these are rarely available. One hundred ten whole genomes of cannabis cultivars, from wild plants and historical varieties to modern hybrids, with a focus on Asian sources to account for the likely domestication origin, were recently sequenced and analyzed to provide an invaluable genetic framework for the history of the plant; the resulting information can be applied to secondary metabolite investigations (Ren et al., 2021). With time, the validity of these hypotheses is sure to be determined thanks to this new genomic information, along with valuable insight into the impressive complexity seen within them.

## Conclusion and Future Prospects

Cannabis was left behind in the agricultural research boom of the last century because of its illegal status in most jurisdictions. While many of the advancements in plant science for a wide range of other species are applicable to cannabis, multiple species-specific traits require dedicated research both to gain fundamental insights and to provide evidence-based data to the growing industry. Since industrial agriculture practices became globally established and genomic studies became possible in the 20^th^ century, researchers have been able to elucidate novel agricultural applications derived from molecular-scale understanding, while cannabis applications remain centered on breeding and environmental conditions; cultivation protocols were largely based on anecdotal rather than scientific evidence. For example, the soybean genome has been unraveled to identify genetic markers related to nematode resistance and this has been exploited to support precise breeding strategies (Kim et al., 2016); meanwhile, the simple taxonomy of cannabis remains controversial (Koren et al., 2020). The cannabis research field is slowly catching up to the level of investigation that is observed for other valuable crop species, with one example being a recent study demonstrating a high-throughput assay using genetic markers to identify sex and chemotype of cannabis germplasm (Toth et al., 2020). However, this study was primarily focused on THC:CBD ratios to determine chemotype and when modeling “total potential cannabinoids” only THC, CBD, CBG, and CBC were included, highlighting the limits of current genetic studies (Toth et al., 2020). Regardless of their limitations, these studies signal the beginning of cannabis truly entering 21^st^ century agricultural research.

Trichomes and essential oils in other plant species have been well characterized in recent decades, and it is important that our understanding of cannabis trichomes reach similar levels of comprehension. The increasingly widespread legalization and public acceptance of cannabis suddenly brings a once-shunned plant into a position of intense interest and high demand in a time of exceptional experimental standards, raising expectations that questions surrounding it be answered much more quickly than for previous crops. Simple breeding and agricultural production techniques for influencing metabolite profiles are not precise nor always consistent, leading to a host of potential complications for both producer and consumer. An example of this complication is the growing medicinal and recreational consumer demand for products with greater THC levels, causing a trend referred to as “lab shopping” that is observed where producers will test their products at several laboratories until they receive the desired cannabinoid concentration analysis for their products (Swider, 2021; Zoorob, 2021). The resulting lack of reliability in the identification might potentially lead to health complications and distrust by those who use cannabis for pain mitigation and as an appetite stimulant/anti-emetic. These issues highlight the need for not just a more reliable and ethical approach to cannabis product quality, but also for methods to reliably tailor metabolite production at the trichome source. New approaches, such as phytomicrobiome manipulation and exploitation, present interesting possibilities, as root inoculums have demonstrated similar effects on THC and CBD contents to nitrogen application (Pagnani et al., 2018; Lyu et al., 2019). If methods can be developed to consistently replicate specific metabolite concentrations and combinations within small ranges across cannabis plants at the trichome level, and if these methods were to become standard across the industry, the benefits for both producers, medical practitioners, and consumers would be great.

From a scientific perspective, multiple interesting questions are associated with the glandular trichomes. Primarily, these questions center around differences related to genotype and growing conditions. How changes to soil composition, light, nutrients, water levels, and other environmental factors affect trichome densities remain largely unknown for cannabis. Our knowledge on how the metabolite profiles themselves differ among varieties is limited and primarily based on poor reporting from growers that are incomplete beyond the major cannabinoids and terpenes, leaving 100 of metabolites unknown. Our lack of knowledge in these areas of cannabis metabolism and composition make it difficult to directly hypothesize exactly where and how differences occur, stressing the need for rigorous uniform standards to allow unbiased and scientifically sound data comparisons. The more we understand about trichomes, the more applicable our knowledge of this plant will be to those along the chain of production and consumption.

## Author Contributions

CT compiled the literature and prepared the manuscript. AG and DS provided the conceptual context. RB, DS, and AG provided the revision and feedback. All authors contributed to the article and approved the submitted version.

## Funding

Funding for this work was provided by the NSERC (CREATE 543319-2020) and the RGPIN/04714-2018, RGPIN-2020-07047.

## Conflict of Interest

The authors declare that the research was conducted in the absence of any commercial or financial relationships that could be construed as a potential conflict of interest.

## Publisher’s Note

All claims expressed in this article are solely those of the authors and do not necessarily represent those of their affiliated organizations, or those of the publisher, the editors and the reviewers. Any product that may be evaluated in this article, or claim that may be made by its manufacturer, is not guaranteed or endorsed by the publisher.
